# Experiences of experts in intelligence measurement of South African school learners

**DOI:** 10.4102/ajopa.v6i0.148

**Published:** 2024-05-20

**Authors:** Ilze van der Merwe, Petro Erasmus, Werner de Klerk

**Affiliations:** 1 School of Psychosocial Health, Faculty of Health Sciences, North-West University, Potchefstroom, South Africa

**Keywords:** experiences, intelligence assessment, development, school learners, South Africa, qualitative

## Abstract

This qualitative research study emerged from the essential need for reliable and valid intelligence test instruments for South African school learners, who are characterised as a diverse population with their variety in culture, ethnicity, and language, as well as having unequal socio-economic and educational backgrounds. The aim of this research study was to use a qualitative interpretive description research design to explore and describe the experiences of both experts in intelligence test development and/or adaptation as well as psychologists and psychometrists who have administered intelligence tests to South African school learners in various contexts. Twelve psychologists and/or psychometrists were interviewed, of which six were also experts in test development and/or adaptation, which yielded four themes after thematic analysis, namely, utilised intelligence measurements in the current South African school learner context are less relevant; the South African education system is a major issue specifically within lower socio-economic status (SES) contexts; it does not seem feasible to design or adapt suitable intelligence measures that are valid and reliable in the current South African school learner context; and key informants’ recommendations from their experiences.

**Contribution:**

This research study contributes to the understanding of the measurement of intelligence of South African school learners in diverse contexts. Findings of this research study can guide the strategic process to design an intelligence instrument suitable for a South African population of school learners, informing fair assessment practices for multiethnic equalisation.

## Introduction

Influenced by the post-apartheid legislation promulgated in the Bill of Rights of South Africa as part of the Constitution (South African Government, 1996), South African practitioners in the field of the psychometric assessment of intelligence of school learners and psychometric instrument adaptation and/or development realised the need and obligation for fair assessment practices (Foxcroft et al., 2004; Laher & Cockcroft, 2014; Meiring, 2007). This would include the administration, creation, or adaptation of fair and unbiased assessment instruments applicable to a multilingual and multiethnic society (Laher & Cockcroft, 2014). The South African school learner population is diverse, not only in terms of culture, ethnicity, and language, but also because of past unequal socio-economic and educational opportunities (Laher & Cockcroft, 2017; Shuttleworth-Edwards, 2016; Van der Merwe et al., 2022). Despite almost three decades of restoration practices in the democratic South Africa, vast discrepancies remain among the school learners (National Planning Commission [NPC], 2020; Ogbonnaya & Awuah, 2019; Van der Berg et al., 2020). There are different population sub-groups based on socio-economic variances, resulting in school learners having unequal access to essential services and disparate levels of quality environments found both at home and in school (Laher & Cockcroft, 2017; NPC, 2020; Ogbonnaya & Awuah, 2019; Van der Berg et al., 2020). Assessing the intelligence of multilingual and multiethnic local school learners from and within various environments poses challenges to both experts developing and/or adapting intelligence instruments, and psychometrists and/or psychologists administering the instruments (Foxcroft, 2004; Laher & Cockcroft, 2017; Lucas, 2013; Van der Merwe et al., 2022). According to the critical review conducted by Van der Merwe et al. (2022), there is no specific reference made to published studies in South Africa on various tests that assess intellectual functioning of South African school learners. The current manuscript then asks the further question regarding what those using these tests say about their experiences.

## Historical changes in the new democratic South Africa

In 2004, a decade after the commencement of the democratic South Africa, the Human Sciences Research Council (HSRC) conducted a comprehensive survey into the test-use patterns and needs of psychological assessment practitioners in South Africa (Foxcroft et al., 2004). Key findings included that many psychometric tests were outdated and not linguistically or cross-culturally relevant, consequently reducing their value, leading to an urgent plea for reliable revision and adjustments (Foxcroft et al., 2004). From the beginning of the democratic South Africa in 1994, a number of normative research projects were launched on differing population groups using various assessment instruments (August, 2017; Laher & Cockcroft, 2017; Laher et al., 2019). According to Van der Merwe et al. (2022), contextual and demographic factors, for example quality of schooling, language, and culture, could affect the school learners’ test performance and consequent intervention plans, which could prove ineffective if the test instrument was outdated and not valid and reliable for the group of learners assessed.

## Present South African intelligence assessment environment

South African psychologists and psychometrists presently have a limited selection of individual intelligence tests for assessing school learners (Mitchell et al., 2018; Van der Merwe et al., 2022). These instruments include the Learning Potential Computerised Adaptive Test (LPCAT; De Beer, 2000), Senior South African Individual Scale–Revised (SSAIS-R; Van Eeden, 1991), and Junior South African Individual Scale (JSAIS; Madge, 1981), which are locally developed tests (Van der Merwe et al., 2022). As a result of the scarcity of such locally developed instruments, South African practitioners frequently need to rely on imported tests that are expensive and not developed to meet the needs of local populations (Laher & Cockcroft, 2017; Shuttleworth-Edwards, 2016). The Raven’s Educational Progressive Matrices and Vocabulary Scales (Raven, 2008), Kaufman Assessment Batteries for Children, 2nd edition (KABC-II; Kaufman & Kaufman, 2004), and the Wechsler Intelligence Scale for Children, 5th edition (WISC-V; Wechsler, 2014) serve as examples of such tests administered in South Africa (Benadé, 2023; Cassoojee, 2020).

The intelligence test instruments commonly administered in South Africa are based on the psychometric model of testing, determining intelligence according to the individual’s ability to perform test tasks and providing an indication of cognitive strengths and weaknesses (Cockcroft, 2013). To conduct such tests effectively as psychologists and/or psychometrists in a new inclusive framework, as promulgated by South African legislative policies, Swart and Pettipher (2005) propose shifting the approach from a deficient-focused assessment (as prescribed by the medical deficit model) towards an asset-based, social systems change approach, where the school learner’s personal strengths (intrinsic qualities) and assets (extrinsic resources in their social environment) are identified and utilised. The psychological assessment practice should be conducted in a dynamic and flexible manner, selecting ‘a battery of assessment measures and procedures applicable to the needs of the specific client and in response to the specific referral question’ (Elkonin et al., 2004, p. 282). This, however, is not entirely possible if most assessment instruments available to these experts are not applicable or outdated (Lucas, 2013; Mitchell et al., 2018; Van der Merwe et al., 2022). Key findings from the critical review by Van der Merwe et al. (2022) reveal the inapplicability of many intelligence instruments when administered to diverse South African school learners. This renders them invalid, inappropriate, and unfair for many school learners. Contextual and demographic influences can impact significantly on test performances, especially if the test was originally designed for and normed using a different population group or developed within a different time frame. This underscores the importance of considering the test instrument’s content, developed norms, and the date of publication before assessing learners (Van der Merwe et al., 2022). Moreover, there is a lack of published studies that cover the topic of applying various tests that appropriately assess intellectual functioning of school learners in South Africa (Van der Merwe et al., 2022).

In light of the above-mentioned statements, this research study aimed to address this gap in the field of psychometry, by collecting data from the experiences of a group of professionals working within the multicontextual domain of the measurement of intelligence of South African school learners. The following research question guided this study: *What are the experiences of intelligence test developing and adaptation experts as well as psychologists and psychometrists who have administered intelligence assessment instruments to South African school learners in various contexts?*

## Methods

### Research design

This research study used a qualitative interpretive description research design to meet the goal of the study. As a smaller, qualitative scale of investigation, the interpretive description method suited this study’s research aim and question well, as it allowed for more in-depth findings and clinical understanding through capturing themes and patterns within subjective, yet reasoned and mindful interpretations (Thorne, 2016) of the experiences of experts, psychologists and psychometrists.

### Sample

Two types of population groups were included in the research study. The first consisted of professional experts with knowledge and experience in developing and/or adapting intelligence tests for reasons such as standardised norming, updating, or adapting. They should have adapted and/or developed at least one intelligence test, as they needed to have experienced this process to provide any information regarding the research topic. Psychologists and psychometrists registered at the Health Professions Council of South Africa (HPCSA) and practising in the field of psychometric testing were chosen as the second group type. They needed to have sufficient experience in conducting intellectual assessments on school learners in various contexts in South Africa. The final sample consisted of 12 psychologists and/or psychometrists, of which six were also experts in test development and/or adaptation. Participation in the research study was voluntary. Participants were diverse in terms of gender, race, type of HPCSA registration and/or field of practice, and number of years practising in their registered fields (see Table 1). As illustrated in Table 2, the participants represented all nine provinces across South Africa. Their experience in the field of psychology and/or psychometry varied. Collectively, the participants have experienced working with school learners from the five mainly found race groups in South Africa, who attend schooling in the three phases found in South African schools, namely Foundation, Intermediate, and Senior Phase. Most of the participants worked with learners from all four levels of SES. Learners’ parents varied in their levels of education received, from having no scholastic background up to having a tertiary level of education (see Table 2).

**TABLE 1:** Demographic information of participants.

Participants’ number	Race	Gender	Field/type of HPCSA registration	Years of practice as registered practitioner	Administered intelligence tests to South African school learners	Adapted and/or developed intelligence test(s)
*P1*	Mixed race	Male	Psychometrist	21–30 years	Yes	No
*P2*	Caucasian	Female	Psychometrist	6–10 years	Yes	No
*P3*	Indian	Female	Psychometrist and Research Psychologist	6–10 years	Yes	Adapted
*P4*	African	Female	Psychometrist	1–5 years	Yes	No
*P5*	African	Female	Educational Psychologist	11–20 years	Yes	Adapted
*P6*	Caucasian	Female	Research Psychologist	More than 30 years	Yes	Developed and Adapted
*P7*	Caucasian	Female	Educational Psychologist	21–30 years	Yes	No
*P8*	Caucasian	Female	Counselling and Neuropsychologist	21–30 years	Yes	Adapted
*P9*	Caucasian	Female	Psychometrist and Research Psychologist	21–30 years	Yes	Developed and Adapted
*P10*	Caucasian	Female	Educational Psychologist	11–20 years	Yes	No
*P11*	Caucasian	Male	Clinical and Industrial Psychologist	More than 30 years	Yes	Developed and Adapted
*P12*	Caucasian	Female	Educational Psychologist	11–20 years	Yes	No

HPCSA, Health Professions Council of South Africa.

**TABLE 2:** Demographic information of learners assessed.

Partici-pant Numbers	Provinces assessed									Learner age/phase school level			Parental education level				Learner race					Learner socio-economic status			
	Gauteng	Mpuma-langa	North West	Limpopo	Free State	Northern Cape	Eastern Cape	Western Cape	KwaZulu-Natal	5–9 years Foundation Phase	9–11 years Inter-mediate Phase	11–20 years Senior Phase	Post-school (tertiary) level	Grade 12 or equivalent	Schooling less than Grade 12	No schooling	Mixed race persons	African persons	Indian persons	Caucasian persons	Asian persons	Privileged socio-economic	Average socio-economic	Lower socio-economic	Very poor socio-economic
*P1*								x		x	x	x	x	x	x	x	x	x	x	x	x	x	x	x	x
*P2*							x		x	x	x	x	x	x	x		x	x	x	x			x	x	
*P3*	x							x				x			x	x		x	x					x	x
*P4*	x									x	x	x	x	x				x				x	x	x	
*P5*	x	x	x	x						x	x	x	x	x	x	x	x	x	x	x	x	x	x	x	x
*P6*	x				x	x	x	x	x			x	x	x	x	x	x	x	x	x	x	x	x	x	x
*P7*	x									x	x	x	x	x	x		x	x	x	x		x	x	x	x
*P8*								x		x	x	x	x	x	x		x	x		x		x	x	x	x
*P9*	x					x		x	x	x	x	x	x	x	x		x	x	x	x	x	x	x	x	x
*P10*	x									x	x	x	x	x				x	x	x		x	x	x	x
*P11*	x							x				x	x	x	x	x	x	x	x	x		x	x	x	x
*P12*					x					x	x	x	x	x	x	x	x	x	x	x	x	x	x	x	x

### Data collection

Data were collected through a demographic questionnaire and semi-structured interviews, using an internet-based communication platform. Semi-structured interviews enabled the primary researcher to present herself in a formal, well-prepared manner, as well as leave room for probing and clarifying questions during the interviews. This led to flexible, interactive, and responsive conversations, allowing for qualitative, meaningful, and in-depth data (Williams, 2019). Questions were asked in the participants’ preferred language (English or Afrikaans; see Table 3 [*English version only*]). Interviews were held until data saturation was reached.

**TABLE 3:** Questions asked during semi-structured interviews.

Questions asked to psychologists and psychometrists	Do you believe the locally administered intelligence tests are applicable to South African school learners from different ethnic, socio-economic and/or academic backgrounds? Kindly motivate your answer. When and for which purpose do you use intelligence tests? Which intelligence test instruments do you use? What is your motivation for selecting this intelligence test instrument(s) above others available in South Africa? Would you recommend any amendments or changes to be made to intelligence tests? If so, which amendments or changes would you recommend? If not, kindly motivate your answer. In which way is the context in which you administer intellectual assessments unique from other contexts? Were there elements in the assessed learners’ contexts that seemed to have acted as barriers or challenges to their test performance? Kindly elaborate. Do you think that the intelligence assessment instrument content considers the school learners’ strengths internally and externally, or is it deficient-focussed? How would you briefly describe your overall experience of administering intellectual assessments on school learners in this unique assessment context?
Questions asked to experts in intelligence test development and/or adaptation	Do you think the administered intelligence tests are applicable to South African school learners from different ethnic, socio-economic and/or academic backgrounds? Kindly motivate your answer. When and for which purpose did you develop/adapt an intelligence test(s)? What were the most significant challenges during the development/adaptation of the intelligence test(s)? What were the most significant insights gained during the development/adaptation of the intelligence test(s)? How would you briefly describe your overall experience of developing/adapting intelligence tests for school learners?

### Data analysis

The interviews held were transcribed verbatim, from which themes were identified through reflexive thematic analysis (Braun & Clarke, 2021; Byrne, 2022), following procedures proposed by Clarke and Braun (2013): (1) familiarisation process by actively reading and re-reading through the data with interpretive reflection; (2) assigning code labels to text segments according to meaning and significance; (3) identifying themes from codes; (4) reviewing themes against the research question; (5) defining and labelling themes; and (6) writing a final report. Coding was independently conducted by the first and third authors, as well as a registered research psychology intern within the School of Psychosocial Health, North-West University, acting as independent, external data coder and analyser to interpret data with a neutral, unbiased perception. This process was followed by a meeting held by the coders and second author, where notes (visual illustrations of each coder’s identified codes and suggested themes) were compared and discussed to determine the final themes and findings.

Taking the data analysis process of constructing and handling data in relation to the research topic to a second, more progressive level, as suggested by the interpretive description design, the researchers aspired to go beyond merely jotting down a list of identified topics. The data analysts employed sound inductive reasoning skills by studying, reflecting, and questioning identified elements interpretively and critically within the context of their relationship to each other, as well as weighing up and confirming findings against the research topic, context, and theoretical knowledge base, as suggested by Thorne (2016).

## Results

Four main themes with sub-themes emerged from the analysed recorded data (see Table 4). To support the themes, applicable verbatim quotations are provided.

**TABLE 4:** Themes and sub-themes.

Themes	Sub-themes
Theme 1: Utilised intelligence measurements in the current South African school learner context are less relevant	1.1 A lack of ongoing research 1.2 Due to multilingual and multicultural contexts 1.3 Outdated items
Theme 2: The South African education system is a major issue specifically within lower socio-economic status contexts	2.1 Many schools within lower socio-economic status contexts in South Africa provide substandard education for school learners 2.2 Should therefore be careful how we interpret results of current intelligence measures
Theme 3: It does not seem feasible to design or adapt intelligence measures that are valid and reliable in the current South African school learner context	-
Theme 4: Key informants’ recommendations from their experiences	4.1 Need to develop our own South African framework 4.2 We need input from all different groups represented in South Africa to really understand how we can uniquely define and conceptualise intelligence 4.3 Need to base use of type of intelligence measurement on context or functioning of the school learner 4.4 Update to reflect modern context 4.5 Appropriate norming needed 4.6 Need to establish optimal contexts and practices during preparation, assessment, and reporting processes

### Theme 1: Utilised intelligence measurements in the current South African school learner context are less relevant

From the collected data, it emerged that the intelligence measurements being administered to South African school learners have become outdated and inapplicable when considering their present, modern, multicultural, and multilingual contexts. The absence of continuous research seemed to be an important explanation.

#### Sub-theme 1.1: A lack of ongoing research

Participants commented on the utilised intelligence instruments being outdated and not applicable in the school learners’ present contexts because ongoing research did not occur. Participant 5 stated:

‘[…] the context of testing was different then. The assessment tools are not adapted to the times that we are in.’ (P5, Female, Educational Psychologist)

Participant 11 referred to the limitations found when conducting research:

‘[…] research is limited […] we don’t have that access any more … the HSRC simply told the schools: “We are coming to you.” Not anymore. We are very reliant on opportunity samples.’ (P11, Male, Clinical and Industrial Psychologist)

Also referring to research limitations, specifically research capacity, Participant 7 argued the lack of novel tests stemming from universities functioning in isolation, with no cooperation with other involved parties:

‘[…] problems stem from our history […] if everyone had stood together, we could have already developed test batteries that hold benefit to our country and community […].’ (P7, Female, Educational Psychologist)

Participant 9 observed:

‘That is where our tests are behind, we do not really have the capacity to do that type of theoretical research to keep ahead and adjust our tests accordingly […] our tests [*have*] become outdated […].’ (P9, Female, Psychometrist and Research Psychologist)

But added that this does not necessarily make the test invalid:

‘[…] something like the SSAIS is criticised, it doesn’t have the latest Carroll Horn theory […] the test becomes more sophisticated, but it doesn’t make that which the SSAIS measures invalid […] That basic information did not disappear.’ (P9, Female, Psychometrist and Research Psychologist)

Hence, it appears that intelligence measurements administered to South African school learners have become less relevant when considering the learners’ current contexts, because continuous research did not happen.

#### Sub-theme 1.2: Due to multilingual and multicultural contexts

Many participants ascribed the unsuitability and inappropriateness of many utilised intelligence instruments to the multilingual and multicultural formation of local school learner groups. Participant 6, responding to whether locally administered tests are applicable to local school learners:

‘Generally, no, and that is the dilemma that we have […] because of our multilingual, multicultural composition of our learners […] impossibility of creating tests that are fair to everybody.’ (P6, Female, Research Psychologist)

She also stated how language could act as a barrier if the test is not administered in the school learner’s first language:

‘[…] the language of test administration, if it’s not the first language of the learner, obviously, that’s a barrier.’ (P6, Female, Research Psychologist)

Some participants stated how tests cannot be translated into the 11 official South African languages because of various existing dialects and obstacles with translations and back-translations. Other participants described how certain concepts of the same language differ, depending on the area in which that language is spoken.

Participant 7 stated how test items do not reflect the local population group or context:

‘[…] it does not reflect their world […] instruments that come from overseas would address the population group in the context where it was created and not the South African context […].’ (P7, Female, Educational Psychologist)

Having school learners from diverse contextual backgrounds, Participant 6 observed the inapplicability of timed test items on South Africans, with elements such as a cultural sense of urgency, which affects how these tasks are undertaken:

‘[…] many external factors […] affect the time and the reaction […] that kind of research has no place in South Africa. It totally misses the point of having diversity of backgrounds, […] languages, the understanding, the cultural sense of urgency […].’ (P6, Female, Research Psychologist)

Some participants found that the role of ethnicity differences between assessor and assessee could also influence assessments.

It would appear that the utilised intelligence instruments are not appropriate for many local school learners, specifically when considering learners’ contextual backgrounds that are linguistically and culturally diverse.

#### Sub-theme 1.3: Outdated items

Participants mentioned that items in intelligence instruments, including language and graphics, were outdated and thus irrelevant to the school learner’s modern world. Participants 8 and 12 explained that certain items in the SSAIS-R and JSAIS are outdated, making them unsuitable to the modern culture:

‘[…] were firstly developed a long time ago […] a lot of the items are outdated […] some are not suitable because it’s not part of our modern culture.’ (P8, Female, Counselling and Neuropsychologist)

‘[…] not relevant anymore […] the pictures and vocabulary […] relevancy is questionable. It’s old-school.’ (P12, Female, Educational Psychologist)

Participant 5 mentioned how the change of context over time has caused incomprehensiveness of test questions:

‘[…] item questions, some of them need a lot of explanation for the child to understand […] as the context has changed.’ (P5, Female, Educational Psychologist)

Outdated language in intelligence measurements was also mentioned, referring specifically to the translated test instruments:

‘Your Zulu, your Setswana, your Sepedi […] the words in those tests are outdated […].’ (P4, Female, Psychometrist)

Participant 7 also noted outdated vocabulary use and test items, arguing that if children were unable to answer these questions, it would not necessarily indicate lower cognitive functioning.

Thus, items found in intelligence tests are outdated, making them unsuitable, less relevant, and thus questionable to administer to local school learners in their modern contexts. Apart from this, as is discussed in the next theme, it seems as though the country’s education system acts as a major contributor to the issues found in intelligence testing.

### Theme 2: The South African education system is a major issue specifically within lower socio-economic status contexts

Lower quality of education provided by schools located in the lower socio-economic status (SES) areas was indicated as contributing to the issue of intelligence instrument inapplicability.

#### Sub-theme 2.1: Many schools within lower socio-economic status contexts in South Africa provide substandard education for school learners

Many schools located in lower socio-economic environments were reported to provide lower quality education. This may act as a hinderance that causes these school learners to perform inadequately on intelligence tests. Participant 12 mentioned:

‘[…] we see that they often function on a low level […] scholastically they show much more handicaps than our kids who might come from another socio-economic group.’ (P12, Female, Educational Psychologist)

Participant 8 noticed the poor quality of schooling offered by schools in disadvantaged areas:

‘“[…] about 90% of them come from a more disadvantaged socio-economic background […] often with very poor quality of schooling […] either in a township or rural area” and, “[…] what I mean by disadvantaged is that the school itself is not well resourced”.’ (P8, Female, Counselling and Neuropsychologist)

Providing examples:

‘[…] they don’t have libraries or computer rooms, often toilets aren’t working properly. Teachers are often not trained all that well and not all that motivated to teach, classes are overfull …’ (P8, Female, Counselling and Neuropsychologist)

Other challenges:

‘“[…] often children walk very far to school and it’s dangerous, so they’re not always motivated to attend school every day […]” also, “[…] parents are often not well educated themselves, so can’t help them with their schooling, don’t really see the need for schooling […]” and, “[…] not really all that involved in their [*children’s*] lives […] a lot of drug abuse, alcohol abuse […]”.’ (P8, Female, Counselling and Neuropsychologist)

In view of the hindering influence of varied educational provision on school learner intellectual test performance, participants commented on how results from these measurements need to be carefully considered.

#### Sub-theme 2.2: Should therefore be careful how we interpret results of current intelligence measures

Considering fair assessment practice and learners’ educational backgrounds, Participant 6 mentioned how school learners’ scores on intelligence tests need to be interpreted cautiously:

‘We must be very careful how we interpret results, unless we can really motivate that it is a fair way of assessing […] and if you look at the educational component […].’ (P6, Female, Research Psychologist)

Participant 5 cautioned that school learners from rural backgrounds may perform below their potential when answering test questions because of limited exposure to the testing language of English:

‘“[…] with your rural and shy learner that does not necessarily mean that they don’t know when they say they don’t know, so you probe a bit more […]” because, “[…] a child who’s not exposed to speaking in English all the way [*speaking English sufficiently*], will answer yes or no when it’s not necessarily the correct answer”.’ (P5, Female, Educational Psychologist)

Because of differing information exposure provided in various environments as well as the verbal element found in intelligence tests, Participant 7 claimed that one cannot infer lower cognitive functioning from underperformance in tests:

‘[…] there’re some of the words in the Vocabulary subtest which children are not exposed to. So, if they cannot answer it, it is not necessarily an indication of lower cognitive functioning, it’s [*rather*] an indication of not being part of their world of living.’ (P7, Female, Educational Psychologist)

Therefore, substandard education provided by many schools within lower SES contexts may act as a major contributor to the school learners’ inadequate performance on intelligence tests, which necessitates the careful interpretation of these school learners’ performance results. Considering the present school environments, it may seem unfeasible to standardise new or adapted intelligence test measurements.

### Theme 3: It does not seem feasible to design or adapt suitable intelligence measures that are valid and reliable in the current South African school learner context

Based upon findings stipulated in themes 1 and 2, attempting to design and adapt valid and reliable tests that will accommodate each and every school learner in the various South African contexts, seemed unfeasible. This perception was supported by Participant 6:

‘It becomes impossible to realistically create a test that will work for everybody […] a fair way of assessing what you then call intelligence.’ (P6, Female, Research Psychologist)

Raising an important question regarding norm collection of multilingual groups:

‘[…] you run into translation difficulties and regional dialects […] can you [*therefore*] focus only on those who have larger than 8% representativity? And can you stick to English only, or can you stick to non-verbal items only?.’ (P6, Female, Research Psychologist)

Calling it an ‘almost unsolvable problem’, Participant 9 stated:

‘“[*N*]orms, this almost unsolvable problem with our heterogeneous population and factors that have the influence on performance […]” and: “[…] aging of norms […] the Flynn-effect says the test should become too easy […] but you cannot just say in twenty years, people become more clever, people [sometimes] become dumber”.’ (P9, Female, Psychometrist and Research Psychologist)

She elaborated:

‘I could use a comparative norm group, but then it would mean that there where I measure poorly, do I then measure equally poorly with all of them?’ (P9, Female, Psychometrist and Research Psychologist)

Providing an example:

‘“[…] a sensible score, let’s say hundred […] it doesn’t mean a hundred. I have [*merely*] measured equally poorly with everyone” because, “[…] if I compare that child with a group where I do measure correctly, then that child falls out. Not because he isn’t hundred, but the external factors play too big of a role”.’ (P9, Female, Psychometrist and Research Psychologist)

Tapping into the shared experience of being part of adaptation projects:

‘[…] my difficulty wasn’t so much the test adaptation […] my colleagues did it amazingly quickly and well. The challenge was to get that back-translated, back to English.’ (P8, Female, Counselling and Neuropsychologist)

Concurring with these difficulties:

‘“[…] the test that I adapted was more for language use and conceptualisation […] significant challenges […] [*were*] staying with the sense of what the question is asking [*keeping to the aim of the question*] while contextualising it […]” because, “[…] a language has different language rules. When you now translate it back, the sense [*test meaning and intent*] is different”.’ (P5, Female, Educational Psychologist)

Participant 8 mentioned how various dialects complicate translation work:

‘[…] you can’t get away from the fact that, no matter what words you use, if the child speaks a different dialect of that language, there might be words that they don’t understand.’ (P8, Female, Counselling and Neuropsychologist)

Participant 9 informed that merely changing test items into African-centred test items does not necessarily change the underlying theoretical construct:

‘“I have attended workshops and congresses where they were going to give me an African-centred test […] I go in very excited and I leave very disappointed […]” because, “… there are [*participant with hands in her hair*] ethnic tests that they say are new, but it is merely the Wechsler-model. There’s nothing new theoretically, there’s nothing new”.’ (P9, Female, Psychometrist and Research Psychologist)

She elaborated that novel and suitable test items merely serve as plasters and do not address the challenge of demographic influences on test performance:

‘“[…] it simply remains the basic thing [*basically the same*]. ‘Instead of two-dimensional, let us use three-dimensional […] and feathers and beads, because it’s more familiar […] or Pattern Completion, but with Venda patterns”, so, “It’s all improvements, but it does not address our basic problem of cross-cultural […] the influence of demographic factors”.’ (P9, Female, Psychometrist and Research Psychologist)

To develop and adapt fair and applicable intelligence measurements to multilingual and multicultural school learners within diverse contexts, tests need to be standardised. Participants found several challenges during this standardisation process:

‘[…] if you calculate norms, you would need a great amount of subgroups, especially in our country […] [*with*] such a heterogenic group, you’re sitting with an extremely large test sample […] Who’s going to test these masses of children?’ (P9, Female, Psychometrist and Research Psychologist)

‘[…] in terms of sample sizes, language, and the use of translations […] that’s a big hurdle […] you want to be representative and have people from all language and culture groups […].’ (P6, Female, Research Psychologist)

‘[…] several colleagues, willing and able to collect norms, taking their own time […] but we still couldn’t get nearly enough […] we use those norms now as a guideline […].’ (P8, Female, Counselling and Neuropsychologist)

‘[…] it would be better if we had much larger sample sizes. We had to limit the ages that we could reach because of limited resources.’ (P8, Female, Counselling and Neuropsychologist)

‘[…] this type of research isn’t cheap […] there aren’t a lot of practitioners in our regions outside of Western Cape, KwaZulu-Natal, and Gauteng […].’ (P3, Female, Psychometrist and Research Psychologist)

‘[…] to really pull representative test samples and get access to it […] to get thousands of profiles means a thousand tests that need to be administered for an hour and a half long individually.’ (P11, Male, Clinical and Industrial Psychologist)

Some participants observed the loss of the government-supported HSRC not performing the role of psychometric test development anymore and questioned the possibility of really being able to collect representative samples.

The above-mentioned challenges suggest that intelligence measurements cannot really be developed or adapted to suit the school learners from diverse areas and educational backgrounds in South Africa. In the next theme, key participants have provided some helpful suggestions of how one could go about still attempting this seemingly impossible task.

### Theme 4: Key informants’ recommendations from their experiences

Derived from their experiences of developing, adapting and/or administering intelligence test batteries to school learners in various regions of South Africa, key participants have shared significant ideas of how to approach the challenging task of attempting to develop and/or adapt intelligence measurements that are fair and applicable.

#### Sub-theme 4.1: Need to develop our own South African framework

Key participants mentioned the need to create a theoretical framework for intelligence testing that is authentic to South Africa, upon which fair and applicable intelligence measurements can be built. Calling it ‘a unique construct’, Participant 6 encouraged designing an original, personalised framework:

‘[…] a unique construct for South Africa […] for cognitive assessment is needed. Where we don’t start with an existing model or understanding of intelligence […] or how it can be measured.’ (P6, Female, Research Psychologist)

Participant 8 suggested:

‘“First ask yourself what you mean by intelligence […] [*what do*] IQ-tests measure […] there’s [*sic*] so many different theories about what IQ is […]”; however, “[…] you can’t only measure a few cognitive functions and generalise that to [*the functioning of*] the whole child […] one’s got to go back to roots of ‘what are you really trying to measure”?’ (P8, Female, Counselling and Neuropsychologist)

Participant 6 explained that it is difficult for South African stakeholders to develop renewed and personalised psychological constructs, because of their inability to move forward from past negative experiences:

‘[…] an international psychology conference linked to the Cross-Cultural Conference […] there was a new body called ‘Forum for African Psychology’ […] I attended to be part of renewal and thinking differently …’ (P6, Female, Research Psychologist)

She continued:

‘“[…] my experience was disappointing […] there was a lot of criticism against what was before and I quite accept that many things were not done with everybody in mind …” however, “[…] it’s easy to say what we never want to see again, but it is not so easy to say: ‘Well, then what should we do”?’ (P6, Female, Research Psychologist)

Despite this, participants still pursued the idea of creating a unique framework, suggesting the representative input of various groups in South Africa.

#### Sub-theme 4.2: We need input from all different groups represented in South Africa to really understand uniquely how we can define and conceptualise intelligence

Stating this sub-theme clearly, Participant 6 suggested:

‘[…] we need to involve people of all cultural and language groups in this journey […] We need input from all different groups represented in South Africa […].’ (P6, Female, Research Psychologist)

Elaborating her point:

‘[…] to really understand uniquely how we can define and conceptualise intelligence […] [*that is*] unique for South Africa, related to the concept of Ubuntu […] linked to their cultural way of being […].’ (P6, Female, Research Psychologist)

Providing practical examples:

‘“We need to go back to the clean slate drawing board and find out how is intelligence experienced, seen, defined, observed, in a multicultural South African context […]” and “[…] from there, try to operationalise this into something measurable and valuable […] in a way that everybody can agree is representative of what is considered intelligent behaviour, or intelligent reasoning”.’ (P6, Female, Research Psychologist)

With tolerance and acceptance, over time, intelligence could be uniquely defined and conceptualised through the multidimensional viewpoints of various South Africans joining forces.

#### Sub-theme 4.3: Need to base use of type of intelligence measurement on context or functioning of the school learner

Key informants recommended selecting administered intelligence tests according to the school learner’s environment or behaviour. Participant 3 expressed this well by stating that:

‘[…] the context in which you’re working, or client with whom you’re working dictates what assessment you can use.’ (P3, Female, Psychometrist and Research Psychologist)

Suggesting that the learners’ background (demographics, context, and type of school) needs consideration during instrument selection:

‘[…] language combined with the age and the type of schooling […] a school in a rural area, township or suburb. So that kind of exposure will decide which tests I will use for which learner.’ (P5, Female, Educational Psychologist)

‘[…] use another instrument which is perhaps friendlier […] the things which could bring challenges – field of experience, terms, language, and culture.’ (P1, Male, Psychometrist)

Participant 3 explained that the assessment aim determines the test being selected:

‘“[…] there’s a range of intelligence assessments […] [*which*] measure different things […] sometimes just a person’s GMA and other times a far more comprehensive, complex understanding of their reasoning [*ability*] […]” therefor, “[…] all of that comes into play in how we select our tools”.’ (P3, Female, Psychometrist and Research Psychologist)

She noticed, however, how practitioners are limited by only being allowed to administer tests recognised by the HPCSA:

‘“Selecting the tools that I use need to be on the HPCSA list […]” however, “[…] sometimes we have assessments […] with [*adequate*] sample size, reliability, validity, non-bias […] differential item functioning and all of those fun things, but it’s not on the HPCSA list, so I can’t use it”.’ (P3, Female, Psychometrist and Research Psychologist)

Reflecting on the above-mentioned statements, it became clear that school learners’ contexts and functioning need to be considered when selecting intelligence instruments for fair assessment. As these learners reside in modern contexts, intelligence instruments should also replicate this essential feature.

#### Sub-theme 4.4: Update to reflect modern context

To represent the modern elements of South African school learners’ contexts, intelligence tests need to be updated. Participant 1 explained that modernising intelligence instruments:

‘“scientifically and statistically” should make them applicable again: “[…] adaptations and renewal […] [*to*] be applicable again […] a new instrument that is scientifically and statistically up to standard”.’ (P1, Male, Psychometrist)

The following participants described how updated versions need to reflect the school learners’ modern contexts:

‘[…] questions could be revised and be made more context-applicable […] more in trend with the child’s life today.’ (P10, Female, Educational Psychologist)

‘[…] the context is very different now […] questions really need to be revised […] contextualised and generalised for all the settings within the country.’ (P5, Female, Educational Psychologist)

Adaptation suggestions:

‘[…] make it more interesting, more creative […] those pictures and illustrations are really quite old-fashioned and come from “long-gone”.’ (P10, Female, Educational Psychologist)

‘“[…] scenarios […] [*should*] not be specific for a certain setting only […] [*as*] a child that is not exposed to certain settings […]” for instance, “[…] an outdated question about a postage and a stamp […] today’s child don’t [*sic*] relate to that. I would rather they be generalised for situations [*which*] children are now exposed [*to*]”.’ (P5, Female, Educational Psychologist)

Participant 2 mentioned updating norms, among other updates:

‘[…] a few updates […] even on norms in South Africa, ‘cause […] our culture and demographics have changed quite considerably.’ (P2, Female, Psychometrist)

Modernising intelligence instruments includes updating norms, which need to be performed in the correct manner.

#### Sub-theme 4.5: Appropriate norming needed

Sharing their experiences in developing, adapting, and/or administering intelligence tests, key informants recommended having adequate norming procedures in place that are designed to promote optimal representation of South African school learners. Participant 5 shared her insights gained during a project of test adaptation:

‘“[…] the variety of people and the context […] when you do the norms, you include almost everybody […]” and the “[…] experience of adapting those tests helped me understand the deeper the importance of norms […] it’s not something that we should take very lightly”.’ (P5, Female, Educational Psychologist)

Participants 9 and 8 highlighted capturing the heterogenic nature of the school learner group to match them demographically:

‘“[…] you standardise to get appropriate norm groups […] especially in our country where you have such a heterogenic group and would want a very heterogenic test sample” therefor, “[…] make provision for everything […] various school types, various socio-economic groups, various language groups, various racial groups”.’ (P9, Female, Psychometrist and Research Psychologist)

‘… [*O*]ne must have enough norms so the child can be matched properly demographically. So, you want to look at age, level of education, quality of education, fluency in a language […].’ (P8, Female, Counselling and Neuropsychologist)

Participant 9 explained how subgroup norming could be applied to heterogenic groups; however, the context needs to have fewer variables:

‘If the population in terms of demographic factors, education, socio-economics, language, etc. is heterogenic […] use subgroup norms, but it only works within very specific environments, the use of within-group norms.’ (P9, Female, Psychometrist and Research Psychologist)

Other than correct norming practices, key participants also recommended the creation of optimal contexts and practices during intelligence assessment.

#### Sub-theme 4.6: Need to establish optimal contexts and practices during preparation, assessment, and reporting processes

Practitioners need to ensure favourable contexts and practices before, during, and after the assessment process. Participant 6 mentioned how practitioners should ensure a fair and appropriate assessment process:

‘[…] psychometrists and psychologists […] must realise that they carry that responsibility […] the total process […] [*should be*] managed appropriately, fairly, and responsibly.’ (P6, Female, Research Psychologist)

Participant 6 further recommended laying the groundwork and getting to know the context first:

‘[…] if you are external to the assessment context, that homework of really understanding the context and getting people to explain it properly to you.’ (P6, Female, Research Psychologist)

She explained how this initial process would entail careful thought and discussions with related parties:

‘[…] that’s the footwork […] [*which*] requires discussions […] taking time and making prior appointments long before the assessment day.’ (P6, Female, Research Psychologist)

If there is resistance, ideas need to be conceptualised that would ensure a fair assessment process:

‘[…] sometimes you get resistance when you communicate with people within a particular context […] [*simply*] put your mind to things that will make it fair in terms of assessment.’ (P6, Female, Research Psychologist)

Participant 9 told of power imbalances that occurred during the apartheid times, where children were merely ‘told’ to be assessed, producing a lot of anxiety:

‘“[…] in the lower socio-economic areas […] it was long ago, so to me it was actually a very, very unfair power relationship there. Those children were very anxious, very defenceless […]” because, “[…] there was an extreme amount of test anxiety. And power imbalance – the school says: ‘You shall be tested now”.’ (P9, Female, Psychometrist and Research Psychologist)

Sadly, Participant 6 has witnessed the same power imbalances still occurring today:

‘Too many times, when you assess in different contexts, you become aware that the people don’t know why they’re there. They were told to be there […].’ (P6, Female, Research Psychologist)

This power play may cause anxiety and underperformance:

‘[…] power dynamics in your authoritative school environments […] from the principal down, you are told: ‘We’re doing assessments today.’

‘[…] there’s not much time for a nice introductory chit-chat to put you at ease […] that’s where the anxiety creeps in and cognitive ability is affected […].’ (P6, Female, Research Psychologist)

To address the stigma that is formed from these unfair practices, Participants 6, 8, and 11 recommended taking time to explain to the school learners of the features and practices involved in the assessment process.

On the day of the assessment, some participants mentioned checking if the children have travelled far before assessment or had enough sleep and nutrition, and attempted to support where possible to provide them an opportunity for best performance. Other than basic needs, Participant 7 noticed the importance of drawing attention to challenges found in school learners’ contexts, which could affect their performance:

‘“I would ask a child: ‘How did you get to school?’” because, for instance, “If a child has driven with an over-anxious, activated, aggressive parent before the assessment, you will sit with a child experiencing anxiousness […] those previous experiences should be taken into account”.’ (P7, Female, Educational Psychologist)

Lastly, some participants recommended that the measurement of intelligence not solely rely on scores derived from the intelligence test, but should include a battery of measures and other information gathered from interviews, observation, and more:

‘We supplement it [*the intelligence test*] […] with additional tests […] considering your four pillars of assessment, you know it includes your interview […] observation […] informal as well as your formal assessment.’ (P7, Female, Educational Psychologist)

‘[…] I never solely rely on the intelligence test […] I consider everything […] I look at those pillars […] ten compare it with the results from the intelligence test […] [*to*] triangulate it [*findings*].’ (P10, Female, Educational Psychologist)

‘[…] it [*the intelligence test*] only serves as a single source of information and [*should be*] viewed in combination with all other information […] for instance […] information from an educator interview.’ (P1, Male, Psychometrist)

‘[…] I do not only administer an IQ-test, […] there’re always other tests added before one can make a final recommendation.’ (P12, Female, Educational Psychologist)

## Discussion

Steered by the research question, the study discovered four main themes (with sub-themes) from the semi-structured interviews with psychologists and/or psychometrists and/or experts who shared their experiences of developing and/or adapting or administering intelligence test instruments to local school learners from and within various contexts. Participants stated how intelligence measuring instruments have become inapplicable when considering the learners’ modern, multicultural, and multilingual contexts, because ongoing research has declined, especially after the HSRC discontinued psychometric test development, and the test items (which include their illustrations and language use) have become outdated. Shortly after the political transition, psychometric research and development by state-funded organisations (such as the HSRC) was halted. By the time new policies for fair testing were implemented (by the *Employment Equity Act 55 of 1998*), many experts had left the country and public sector, and such services were discontinued, causing a substantial decrease in psychometric test development and adaptation (Foxcroft et al., 2004; Tredoux, 2013). Presently, intelligence test instruments have become outdated, inapplicable, and unfair to administer to multicultural and multilingual school learners within various contexts (Laher et al., 2019; Shuttleworth-Edwards, 2023; Van der Merwe et al., 2022); however, these tests are ‘more reliable and valid than any of the limited number of alternatives’ (Laher & Cockcroft, 2013, p. 4). Mitchell et al. (2018) mention the shortage of available culturally appropriate cognitive tests for school learners. This places practitioners in the field of psychometric testing in a challenging predicament, as they are legally and ethically bound to conduct fair and valid testing for all (HPCSA, 2016; ITC, 2015; South African Government, 1996).

Many participants indicated that the unsuitability of intelligence tests is owed to the substandard education provided, specifically by schools located within lower SES contexts, which affect the ability and performance of school learners from these schools. Careful interpretation of the school learners’ test scores and performances was accordingly argued, acknowledging differing exposures to language and information. This agrees with Shuttleworth-Edwards et al. (2013), who stated how the quality of education received by local school learners is related to their performance on intelligence tests. Various South African normative studies posited that considerable differences in intelligence test scores of school learners were not primarily because of ethnic differences, but rather the differences in the quality of education (Amod, 2013; Greenop et al., 2013; Shuttleworth-Edwards et al., 2013). Clearly, South African schools provide various levels in quality of education when comparing the five quintiles into which schools are categorised, according to their SES (Maistry & Africa, 2020; Ogbonnaya & Awuah, 2019) (see quintile and poverty distribution across provinces in DBE [2021]). School learners who go to schools located in disadvantaged areas (quintiles one to three) tend to receive lower quality of education and yield lower academic performances than school learners who attend more privileged schools (quintiles four and five) (Graven, 2014; Ogbonnaya & Awuah, 2019; Spaull, 2012). Inequalities in education provided across various schools have been noticed (see Spaull & Jansen 2019). Referring to the SACMEQ III dataset for South Africa, Spaull (2012) remarked that a school’s SES had a far greater impact on school learner performance than individual learner’s socio-economic background. In addition, Mitchell et al. (2018, p. 435) have drawn attention to how the locally developed intelligence test battery, namely the SSAIS-R, is ‘somewhat biased to educational exposure, in particular verbal and linguistic abilities’, which further supports the argument for unfair, inapplicable intelligence measuring instruments where careful interpretation of intelligence measured results is needed, especially of school learners who attend schools within lower socio-economic contexts (Laher et al., 2019; Mitchell et al., 2018; Shuttleworth-Edwards, 2023).

Guided by the Constitution (South African Government, 1996), as well as guidelines provided by the HPCSA (HPCSA, 2016) and the International Test Commission (ITC, 2015), practitioners in the field of psychometric testing are legally, ethically, and professionally obligated to not only administer measurements that are valid, reliable, and fair to all but also to not withhold services because of reasons of ethnicity, language, culture, among others. As participants regarded the heterogenic nature of the local school learners with their differing contexts, it seemed unrealistic that newly or adapted intelligence measures could be applicable to all South African learners. Examples of challenges include translation difficulties (particularly with multiple South African languages with different vocabularies and language rules to English, and additionally the existence of various regional dialects), issues with test norming and standardisation practices within multiethnic population groups, as well as limited research opportunities because of a lack of resources and accessibility. Questions and concerns were raised around topics such as the influence of demographics and the Flynn-effects during norm development, the possibility of conducting fair and appropriate comparative norming practices within multidimensional population groups, and the feasibility of developing novel, African-centred intelligence tests. Standardisation challenges and concerns were also raised by authors such as Aston (2006) and Truter et al. (2018), who have mentioned how the population-based projects of standardising the Wechsler’s scales (third and fourth editions) for the whole South African population, have received criticism regarding the norming processes followed. Shuttleworth-Edwards (2016, 2017) states that practices of country-wide unitarity norming applied on intelligence tests in countries with culturally heterogenous contexts are invalid, as South Africa does not represent one unified population culture that could be generalised. Shuttleworth-Edwards and Truter (2023) present compelling arguments concerning the practical utilisation of context-specific, within-group norms (in contrast to population-group norms) within a diverse, heterogeneous population across various contexts. Laher and Cockcroft (2014) highlight the positive strides made in developing (e.g., LPCAT), adapting, norming, and standardising psychological test instruments suitable for local contexts; however, they highlighted the need for developing emic measurements that are appropriate for the local African population for whom Western-developed tests may be inapplicable.

Intelligence tests and instruments still function as essential means towards school learner guidance and support, where the combination of interpreted measured cognitive abilities (e.g., their strengths and challenges) and other findings (e.g., learner background information or observations from testing) aid the practitioner in developing applicable intervention strategies (Beal et al., 2019). Despite the former concerns and challenges regarding fair intelligence testing of local school learners, participants have shared and provided insightful suggestions of how one could undertake this seemingly unfeasible task. Recommendations included gathering input from various local groups to define and conceptualise the term ‘intelligence’, designing an original, personalised South African framework, aligning the intelligence measurement to the school learners’ context and functioning, updating the intelligence instrument to reflect the learners’ contexts, norming the intelligence tests adequately, as well as ensuring optimal contexts and practices while preparing, assessing and reporting intelligence measurements of local school learners. Various research studies have been conducted over the past two decades across South Africa (Laher et al., 2019; Shuttleworth-Edwards, 2023). Good examples can be found in the editorial work by Laher and Cockcroft (2013) and in the book by Shuttleworth-Edwards and Truter (2023). Although occurring in separate clusters (various small-scale research projects conducted using smaller population groups), these studies have proved to change values in the field of psychological assessment with attempts of adapting and designing tests that are unbiased, applicable, and fair to the diverse South African population (Laher & Cockcroft, 2014). One could also learn from work that was carried in other developing countries that face similar challenges like South Africa, for instance culturally fair intellectual testing studies conducted by Lozano-Ruiz et al. (2021) in Morocco and Gonthier (2022), who compare results from Western populations to non-Western populations. In addition, following the example of test instruments such as the latest WISC-V edition, practitioners need to steer away from the singular concept of intelligence or general mental ability score and consider intellectual ability rather as a constellation of cognitive abilities (Beal et al., 2019; Kaufman et al., 2016). Despite positive contributions of newly developed and adapted intelligence measurements, the need to have intelligence measurement instruments that will cater for all remains a pressing reality, calling for effective and emic research studies on intelligence instruments (development, adaptation, and norming) and intelligence assessment (Laher & Cockcroft, 2014, 2017; Van der Merwe et al., 2022).

### Limitations and recommendations

The research study’s findings were affected by some limitations. As common for qualitative studies, the sample size was relatively small to allow for more in-depth and mindful interpretations and findings, as suggested by Thorne (2016). Even though each province in South Africa was represented, shared experiences from other practitioners would yield more insight into the topic. A similar research with a larger sample of professionals in the field is recommended. A complementary study of gathering data from the experiences of school learners, parents and educators regarding intelligence testing could additionally yield valuable information.

## Conclusion

Research findings provided significant evidence that many of the intelligence measurement instruments administered to local school learners have been inapplicable and unfair, especially in the view of the multidimensionality of school learners’ demographics, ethnicity, and contexts. Test performance of school learners in schools located in lower socio-economic environments needs to be interpreted with caution. These schools often provide substandard education, and many intelligence test batteries have faced criticism for being biased towards individuals with less educational exposure (Mitchell et al., 2018). Despite numerous challenges, key informants offered recommendations based on their experiences in intelligence testing and/or intellectual instrument adaptation and/or development. Their recommendations aimed to address the difficult task of creating, adapting, and administering intelligence measurements that ensure fairness and applicability to all South African school learners. It is imperative that all South African school learners be provided the opportunity to be intellectually assessed by suitable and ethically fair test instruments, in order to reap the benefits and receive the opportunities, which intellectual assessment can deliver. Such benefits include identification of learning challenges, giftedness, and other important information from which school placement, referrals, and learner support plans could be generated (Beal et al., 2019; Kranzler et al., 2020).
